# Efficacy of once-daily indacaterol 75 μg relative to alternative bronchodilators in COPD: A study level and a patient level network meta-analysis

**DOI:** 10.1186/1471-2466-12-29

**Published:** 2012-06-25

**Authors:** Shannon Cope, Jie Zhang, James Williams, Jeroen P Jansen

**Affiliations:** 1 Mapi Consultancy, Boston, MA, USA; 2 Novartis Pharmaceuticals, Skillman, NJ, USA

## Abstract

**Background:**

The objective of this study was to evaluate the comparative efficacy of indacaterol 75 μg once daily (OD), tiotropium 18 μg OD, salmeterol 50 μg twice daily (BID), formoterol 12 μg BID, and placebo for the treatment of chronic obstructive pulmonary disease (COPD) based on individual patient data (IPD) from randomized controlled trials (RCTs) from the indacaterol trial program and aggregate data (AD) identified from a systematic review of RCTs.

**Methods:**

22 RCTs were included in the AD analysis that evaluated: indacaterol 75 μg (n = 2 studies), indacaterol 150 μg n = 5 (i.e. salmeterol 50 μg) (n = 5), indacaterol 300 μg (n = 2), tiotropium 18 μg (n = 10), salmeterol 50 μg (n = 7), and formoterol 12 μg (n = 4). All of the studies except for one head-to-head comparison (tiotropium vs. salmeterol) were placebo controlled. Outcomes of interest were trough forced expiratory volume in 1 second (FEV_1_) and St. George’s Respiratory Questionnaire (SGRQ) total score at week 12. The AD from all trials was analysed simultaneously using a Bayesian network meta-analysis (NMA) and relative treatment effects between all regimens were obtained. In a separate analysis, the IPD available from the 6 indacaterol RCTs was analysed in a NMA. Treatment-by-covariate interactions were included in both analyses to improve similarity of the trials.

**Results:**

All interventions compared were more efficacious than placebo regarding FEV_1_ at 12 weeks. Indacaterol 75 μg is expected to result in a comparable FEV_1_ at 12 weeks to tiotropium and salmeterol based on both IPD and AD analyses. In comparison to formoterol, the IPD and AD results indicate indacaterol 75 μg is more efficacious (IPD = 0.07 L difference; 95%Credible Interval (CrI) 0.02 to 0.11; AD = 0.05 L difference; 95%CrI 0.01; 0.09). In terms of SGRQ total score at 12 weeks, indacaterol 75 μg and formoterol were more efficacious than placebo, whereas for tiotropium and salmeterol the credible intervals included zero for the AD results only (tiotropium: -2.99 points improvement versus placebo; 95%CrI −6.48 to 0.43; salmeterol:-2.52; 95%CrI: -5.34; 0.44). Both IPD and AD results suggest that indacaterol 75 μg is expected to be comparable to all active treatments.

**Conclusions:**

Based on a synthesis of currently available AD RCT evidence as well as an IPD network meta-analysis of six RCTs, indacaterol 75 μg is expected to be at least as efficacious as formoterol and comparable to tiotropium and salmeterol regarding FEV_1_. Furthermore, indacaterol 75 μg shows comparable level of improvement in health-related quality of life to tiotropium, salmeterol, and formoterol, as measured by the SGRQ.

## Background

Chronic obstructive pulmonary disease (COPD) is a lung disease characterized by airflow limitation which is not fully reversible, involving breathlessness, decreased exercise capacity, and in some cases chronic cough as well as sputum production. Given the progressive nature of the disease, the aim of treatments is to reduce symptoms and exacerbations, thereby improving health-related quality of life. Initially patients are recommended to receive a short-acting bronchodilator (i.e. salbuterol). Once the disease progresses regular treatment with one or more long-acting β2-agonists (LABA) (i.e. indacaterol, salmeterol, or formoterol) or long acting anticholinergic (LAMA) (i.e. tiotropium) is recommended [1].

Indacaterol is a novel once-daily (OD) treatment that provides fast-acting and sustained bronchodilation for patients with moderate to severe COPD. In the United States indacaterol 75 μg has recently been approved by the Food and Drug Administration as a long-term maintenance treatment of airflow obstruction in moderate to severe COPD, while indacaterol 150 μg and 300 μg were approved in 2010 by the European Medicines Agency. Currently there is no randomized clinical trial (RCT) that simultaneously compares all the recommended long-acting maintenance monotherapy treatments. Therefore, in the absence of such an RCT, there is a need for a network meta-analysis to assess the comparative efficacy of indacaterol 75 μg versus tiotropium, salmeterol, and formoterol.

Although mixed treatment comparisons have been published in the area of COPD [2,3], recent studies evaluating indacaterol have not been captured. Moreover, previous analyses relied on study-level or aggregate level data (AD). Since randomization of patients does not hold across trials in a network of RCTs, there might be an imbalance in study and patient characteristics across comparisons possibly causing biased treatment effect estimates. However, with AD one cannot separate within-study associations from across-study associations, and network meta-analysis with AD might be prone to residual confounding bias due to differences in patient characteristics across comparisons [4,5]. In contrast, the current study also includes a network-meta analysis incorporating individual patient data (IPD) from the indacaterol trial program, which allows for meta-regression models to accurately assess heterogeneity due to patient characteristics with adequate power [4,5]. The IPD network meta-analysis is based on six studies from an extensive clinical trial program, including the following trials: B23354 [6], B2355 [7], INVOLVE [8], INHANCE [9], INLIGHT-2 [10], INLIGHT-1 [11]. Results of this analysis are also compared with an AD analysis, which incorporates study-level evidence identified from a systematic review.

The objective of the current study was to estimate the comparative efficacy of indacaterol 75 μg OD, tiotropium 18 μg OD, salmeterol 50 μg twice daily (BID), formoterol 12 μg BID, and placebo in terms of trough forced expiratory volume in 1 second (FEV_1_) and St. George’s Respiratory Questionnaire (SGRQ) total score.

## Methods

### Evidence base

A systematic literature review was performed in order to identify RCTs evaluating the efficacy of indacaterol 75 μg, 150 μg, 300 μg, tiotropium 18 μg, salmeterol 50 μg, and formoterol 12 μg for COPD. MEDLINE® and EMBASE® databases were searched simultaneously for the period of 1989 to 2010. The search strategy has been previously published [12], which includes search terms involving a combination of free-text and thesaurus terms relevant to COPD, indacaterol, salmeterol, formoterol, tiotropium, and RCTs. The relevance of each citation identified from the databases was based on title and abstract according to predefined selection criteria. Study selection criteria in terms of population, outcomes and study design were defined and dictated which articles were selected:

*Population*: Adults with COPD.

*Interventions*: Indacaterol 75/150/300 μg OD, tiotropium 18 μg OD, salmeterol 50 μg BID, and formoterol 12 μg BID.

*Comparators*: Comparators included any of the interventions or placebo.

*Outcomes*: Trough FEV_1_ and SGRQ total score.

*Study Design*: RCTs.

Additionally, any unpublished studies in the indacaterol trial program that evaluated indacaterol 75 μg at 12 weeks were included. Studies within the program based on an Asian population were excluded, as were unpublished supplementary trials. Individual patient data (IPD) was available from the indacaterol RCTs that formed the evidence network for the network meta-analysis (B2354 B23354 [6], B2355 [7], INVOLVE [8], INHANCE [9], INLIGHT-2 [10], INLIGHT-1 [11]). Please note that data for indacaterol 150 and 300 μg was included in the evidence base, although results are not presented for these doses as it was not considered relevant for the current decision problem. Data for indacaterol 600 μg was available from the INVOLVE study which was excluded from the evidence base as this dose is not approved.

For the abstracts that potentially met these criteria, publications were obtained if available. Based on these full text reports, two reviewers evaluated whether each study met the selection criteria. Information was extracted relating to the study design, population characteristics, interventions, and the outcomes of interest at 12 weeks. The difference in the change from baseline (CFB) or least square mean outcome at follow-up was extracted for each outcome of interest or was calculated as the difference between the CFB (or least square mean at follow-up) for the active and placebo treatments. If necessary, the outcomes of interest were extracted from Figures using DigitizIt software version 1.5.8. The standard error of the difference in CFB was extracted where available or calculated based on the available uncertainty estimates reported. The standard error of the difference was imputed where necessary based on the average standard deviation across the included studies and combined with the study-specific sample size.

### Outcomes of interest

The outcomes of interest were: FEV_1_ at 24 hrs post-dose (‘trough’; mean of the values assessed at 23 h 10 min and 23 h 45 min following the previous morning dose), and health status as assessed by the SGRQ total score at 12 weeks. FEV_1_ was selected as it represented the primary outcome in all six indacaterol trials, while SGRQ total score reflects a patient reported outcome measured by a validated instrument.

### Analysis

For each of the endpoints of interest a Bayesian network meta-analysis was performed [13,14] in two separate analyses using 1) IPD from the indacaterol studies and 2) AD from all studies. Analyses within the Bayesian framework involve data, a likelihood distribution, parameters, a model, and a prior distribution [15]. The model relates the data from the individual studies to the basic parameters in order to estimate the relative treatment effects of each intervention compared to placebo [16]. The relative efficacy estimates between all the interventions can be calculated as a function of the basic parameters for each intervention [16].

In order to minimize confounding bias, treatment-by-covariate interactions were incorporated in the models [17]. Potential effect modifies were selected based on clinical expertise. Therefore, in addition to treatment and study effects, the final models analysed the outcome at 12 weeks including covariates and treatment by covariate interactions. The IPD analysis included the following covariates: baseline value of outcome, proportion of current smokers (as opposed to ex-smokers), reversibility to short-acting β2-agonists, and reversibility to short-acting anticholinergic. Since reversibility data was not consistently reported at the study level, the following covariates were included in the AD analysis: proportion of current smokers, proportion of patients with severe or very severe COPD (as opposed to mild or moderate COPD) based on the GOLD guidelines; average age, and proportion of males. In the case of the AD analyses, the CFB was analysed and therefore the baseline values of the outcomes were not included as covariates as was done for the IPD, where the outcome at 12 weeks follow-up was analyzed. Analyses without adjustment for covariates were also performed.

Linear models with normal likelihood distributions were used. To minimize the influence of prior beliefs on the analysis, all model parameters were estimated using non-informative prior distributions. Sensitivity analyses were performed on the prior distribution by varying the level of precision. The deviance information criterion (DIC), which provides a measure of model fit that penalizes model complexity, was used to select fixed effects models over random effects models [15,18,19].

All models were analyzed using Markov Chain Monte Carlo techniques (MCMC) with WinBUGS 1.4.1. The IPD was programmed using R (Version 2.8.1). For each analysis the Gelman-Rubin statistic (as modified by Brooks and Gelman [20]) was visually inspected based on a graphical plot of the starting iteration of each range illustrating the approximate point of convergence. The posterior distribution for the relative efficacy of indacaterol 75 μg compared to the treatment alternatives (i.e. difference in FEV_1_ or SGRQ total score) were summarized with the median as measure of the point estimate and the 2.5th and 97.5th percentile to reflect the 95 % Credible Interval (CrI). 95 % CrIs represent the 95 % probability that the true underlying effect lies in the interval specified. The probability that indacaterol 75 μg was better than the alternatives is also presented.

## Results

### Study and patient characteristics

**Figure 1:**
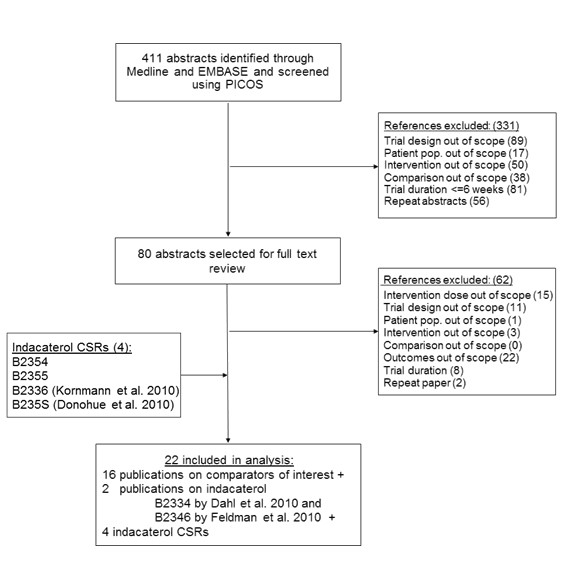
Flow Diagram of study selection.

The study selection process is summarized in Figure 1. The literature search identified 411 potentially relevant studies. The first review excluded 331 (81 %) of potentially relevant studies which did not meet selection criteria. The reasons for exclusion were related to the following factors: trial design – 89 (22 %), trial duration was less than or equal to 6 weeks – 81 (20 %), intervention – 50 (12 %), duplication – 56 (14 %), comparator – 38 (9 %), and population – 17 (4 %). The full text review of 80 remaining studies excluded 62 (15 %) studies for reasons including outcomes– 22 (5 %), intervention dose out of scope– 15 (4 %), study design – 11 (3 %), trial duration– 8 (2 %), intervention– 3 (1 %), population– 1 (<1 %), and duplication– 2 (<1 %). Overall, 18 publications were identified from the search of the databases, which included two indacaterol publications by Dahl et al. 2010 for study B2334 and by Feldman et al. 2010 for study B2346. Two additional RCTs assessing indacaterol 75 μg were provided by Novartis (clinical trial reports for B2354, and B2355), as well as 2 RCTs evaluating indacaterol 150 and 300 μg which were published at the time of the analysis (B2335S by Donohue, 2010; B2336 by Kornmann 2010). Therefore, overall there were 6 indacaterol studies included. In total, 22 RCTs were included in the AD analysis [21-36].

**Figure 2:**
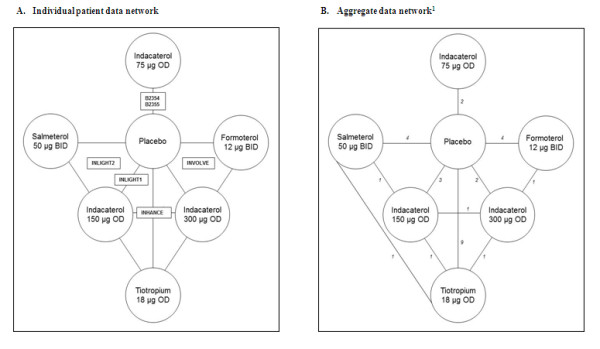
** Network of evidence.**^1^Note: Two 3-arm trials and one 4-arm trial were included and counted separately; therefore totals do not sum to 22.

In Figure 2 the network of RCTs is presented. Figure 2A illustrates the trials where IPD was available and Figure 2B presents the network containing all studies where study level AD was used. In the IPD network of studies B2354 and B2355 assessed indacaterol 75 μg versus placebo. The INVOLVE study evaluated indacaterol 300 μg and 600 μg OD compared to placebo and formoterol 12 μg BID over 52 weeks. INHANCE assessed indacaterol 150 μg and 300 μg OD compared to placebo and tiotropium 18 μg OD over 26 weeks. INLIGHT-2 compared indacaterol 150 μg OD to placebo as well as salmeterol 50 μg BID over 26 weeks, and INLIGHT-1 evaluated indacaterol 150 μg OD compared to placebo over 12 weeks. When the network was extended to include the AD from the studies identified in the systematic review, 16 studies were added to the evidence base for the comparisons of tiotropium, salmeterol, and formoterol versus placebo as well as one study directly comparing tiotropium to salmeterol. The included studies were multicentre parallel RCTs that included a placebo arm, except for the head to head study by Briggs et al. 2005. All treatments were administered in a double-blind fashion (except for the open-label tiotropium arm in the INHANCE study) and the studies were performed predominantly in Europe and North America.

Details of trial designs and characteristics of patients included in the studies are provided in Table 1 and Table 2. The enrolled patients were adults with a COPD diagnosis. Included patients were 40 years of age or older who were most often required to have an FEV_1_/Forced Vital Capacity (FVC) of less than or equal to 0.70 and FEV_1_ percent predicted ranging from less than 80 % to less than 50 %. All patients were permitted a short-acting beta-agonist as needed, although there were some differences in other concomitant medications allowed during the trial. For the indacaterol trials, patients using fixed dose combinations of β2-agonists and inhaled corticosteroids (ICS) were switched to equivalent ICS monotherapy (at a dose and regimen to remain consistent throughout the study). Most studies excluded patients that had recently experienced an exacerbation or used health care resources that would suggest an exacerbation (hospitalization, oral corticosteroids), or did not report any specific exacerbation criteria. For example, the indacaterol trials excluded patients with a hospitalization 6 weeks prior to the trial or during run-in period. In contrast, the study by Chan et al., 2007 required patients to have experienced at least 1 exacerbation in the previous year but not in the 6 weeks prior to the trial [34]. Overall, very few studies reported the exacerbation history.

**Table 1:** Key study characteristics for all studies

**Source**	**Trial Design**^**1**^	**Arm 1**	**Centres/Countries**^**2**^	**Inclusion criteria**^**3**^	**Background treatment allowed**^**4**^	**Background treatment not allowed**^**4**^
MOITA, 2008	12 week RCT, PC, DB, MC	Tiotropium; 18 μg; OD (n = 147) vs. Placebo (n = 164)	31 centres/ Portugal	FEV1 ≤ 70 %; FEV1/FVC ≤ 70 %; excluded if ≥ 3 exacerbations previous year	LABAs, theophylline, mucolytics, ICS, stable doses oral corticosteroids. Temporary increases in theophylline or oral steroids for exacerbations	Theophylline 24 h preparations
VERKINDRE, 2006	12 week RCT, PC, DB, MC	Tiotropium; 18 μg; OD (n = 46) vs. Placebo (n = 54)	10 centres/ France	FEV1 ≤ 50 %; FEV1/SVC ≤ 70 %; residual volume ≥ 125 %; excluded if unstable doses oral corticosteroid 6 wks prior	Stable doses oral corticosteroids, ICS, theophylline preparations, mucolytic agents	Use of SABAs, oral ß2-agonists, or LABAs
COVELLI, 2005	12 week RCT, PC, DB, MC	Tiotropium; 18 μg; OD (n = 100) vs. Placebo (n = 96)	12 centres/ USA	FEV1 ≤ 60 %; FEV1/FVC ≤ 70 %; excluded if exacerbation in prior 6 wks	ICS, LABAs and theophyllines	Cromones, leukotriene antagonists, and inhaled anticholinergics
CASABURI, 2000	13 week RCT, PC, DB, MC	Tiotropium; 18 μg; O (n = 279) vs. Placebo (n = 191)	25 centres/ USA	FEV1 ≤ 65 %; FEV1/FVC ≤ 70 %	Stable doses of theophylline, ICS, oral prednisone	Other inhaled or oral bronchodilators
CASABURI, 2002	Two 56 week RCTs, PC, DB, MC	Tiotropium; 18 μg; OD (n = 550) vs. Placebo (n = 371)	50 centers/ countries NR	FEV1 ≤ 65 %; FEV1/FVC ≤ 70 %;	Stable doses of theophylline, ICS, oral prednisone	NR
NIEWOEHNER, 2005	24 week RCT, PC, DB, MC	Tiotropium; 18 μg; OD (n = 914) vs. Placebo (n = 915)	26 centers/ USA	FEV1 ≤ 60 %; FEV1/FVC ≤ 70 %; excluded if not recovered from exacerbation ≥ 30 days prior	All other respiratory medications (including ICS and LABAs)	Open-label anticholinergic bronchodilator
CHAN, 2007	48 week RCT, PC, DB, MC	Tiotropium; 18 μg, OD (n = 608) vs. Placebo (n = 305)	101 centers/ Canada	FEV1 ≤ 65 %; FEV1/FVC ≤ 70 %; included if ≥ 1 exacerbation previous year but not in 6 weeks prior	Stable dose oral corticosteroids, ICS, theophylline preparations, mucolytic preparations (not containing bronchodilators), LABAs	NR
TONNEL, 2008	36 week RCT, PC, DB, MC	Tiotropium; 18 μg: OD (n = 266) vs. Placebo (n = 288)	123 centers/ France	FEV1 20-70 %; FEV1/FVC ≤ 70 %;	Stable doses of theophylline preparations (excluding 24-hour preparations), mucolytics, ICS, and oral steroids	NR
HANANIA, 2003	24 week RCT, PC, DB, MC	Salmeterol; 50 μg; BID (n = 177) vs. Placebo (n = 185)	76 centres/ USA	FEV1 >40 % and <65 %; FEV1/FVC < 70 %; symptoms criteria; excluded if oral corticosteroids 6 wks prior	Stable regimen of theophylline	All other corticosteroids and bronchodilators
MAHLER, 2002	24 week RCT, PC, DB, MC, DD	Salmeterol; 50 μg; BID (n = 160) vs. Placebo (n = 181)	65 centers/ countries NR	FEV1 <65 % but >70 L. FEV1/FVC ≤70 %; excluded if moderate or severe exacerbation during run-in	Theophylline	Corticosteroids and other bronchodilators
VAN RUTTEN, 1999	12 week RCT, PC, DB, MC, DD	Salmeterol; 50 μg; BID (n = 47) vs. Placebo (n = 50)	3 centers/ Netherlands	FEV1 ≥40 % and ≤65 %; FEV1/FVC < 60 % (post salbutamol); symptoms criteria;	Stable doses of maintenance drugs	NR
CELLI, 2003	12 week RCT, PC, DB, MC, DD	Salmeterol; 50 μg; BID (n = 554) vs. Placebo (n = 271)	189 centres/ 15 countries	FEV1 20-70 %; FEV1/FVC < 65 %; <15 % reversibility FEV1; symptom criteria; excluded if exacerbation 6 wks prior	Usual medications at stable dose	β2-adrenoceptor agonists, anticholinergics, antibiotics for respiratory tract infection, leukotriene antagonists
GROSS, 2008	12 week RCT, PC, DB, DD, MC	Formoterol; 12 μg; BID (n = 114) vs. Placebo (n = 114)	38 centres/ USA	FEV1 >30 %; FEV1/FVC < 70 %; symptom criteria; excluded if exacerbation in 4 wks prior	Stable doses of inhaled or oral corticosteroids	NR
ROSSI, 2002	12 month RCT, PC,DB,MC	Formoterol; 12 μg; BID (n = 211) vs. Placebo (n = 220)	81 centers worldwide	FEV1 < 70 % of the predicted value and ≥ 0.75 L, FEV1 vital capacity ratio of <88 % of that predicted in men and <89 % in women.	Inhaled salbutamol (100 microgram per puff) or equivalent doses of albuterol in US centers as needed	NR
DAHL, 2001	12 week RCT, PC, DB, DD, MC	Formoterol 12 μg; BID (n = 194) vs. Placebo (n = 200)	57 centres/ Europe, Russia, Canada, USA	FEV1 <70 %; FEV1/FVC < 88 % for men and <89 % for women; symptom criteria; excluded if used oral corticosteroids 4 wks prior	Stable ICS, short courses of antibiotics, oral corticosteroids, and/or oxygen in case of exacerbation or respiratory infection	NR
BRIGGS, 2005	12 week RCT, DB, MC	Tiotropium 18 μg; OD (n = 328) vs. Salmeterol; 50 μg; BID (n = 325)	50 centres/ Europe, UK and USA	FEV1 ≤ 60 %; FEV1/FVC ≤ 70 %; excluded if exacerbation 4 wks prior	All usual medications	LABAs different from study medication
B2334, 2008/ DAHL, 2010	52 week RCT, PC, DB, MC, DD	Indacaterol; 300 μg; OD (n = 437) vs. Formoterol; 12 μg; BID (n = 435) vs. Placebo (n = 432)	# centres NR/ 25 countries in S. America, Europe, Russia, Africa, and Asia	FEV1 ≥ 30 % and <80 %; FEV1/FVC < 70 %; reversible and non-reversible patients included; excluded if hospitalisation 6 wks prior to trial or during run-in period	ICS monotherapy	Tiotropium, short acting anti-cholinergics, fixed combinations of β2-agonists and ICS or β 2-agonists and inhaled anticholinergics, LABAs, and other SABAs, theophylline, other xanthines, parenteral or oral corticosteroids
B2335S, 2008/ DONOHUE, 2010	26 week RCT, PC, DB (except for tiotropium arm), MC, DD; Adaptive seamless	Indacaterol; 150 μg; OD (n = 420) vs. Indacaterol; 300 μg; OD (n = 418) vs. Tiotropium; 18 μg; OD (n = 410) vs. Placebo (n = 425)	# centres NR/ Argentina, Canada, Europe, India, Italy, Korea, Taiwan, USA	FEV1 ≥ 30 % and <80 %; FEV1/FVC < 70 %; reversible and non-reversible patients included; excluded if hospitalisation 6 wks prior	ICS monotherapy	Tiotropium, short acting anti-cholinergics, fixed combinations of β2-agonists and ICS or β 2-agonists and inhaled anticholinergics, LABAs, and other SABAs, theophylline, other xanthines, parenteral or oral corticosteroids
B2336, 2009/ KORNMANN, 2010	26 week RCT, PC, DB, MC, DD	Indacaterol; 150 μg; OD (n = 333) vs. Salmeterol; 50 μg; BID (n = 334) vs. Placebo (n = 335)	# centres NR/ Canada, Colombia, Europe and Russia, Slovakia, India, Peru, Taiwan	FEV1 ≥ 30 % and <80 %; FEV1/FVC < 70 %; reversible and non-reversible patients included; excluded if hospitalisation 6 wks prior	ICS monotherapy	Tiotropium, short acting anti-cholinergics, fixed combinations of β2-agonists + ICS or β 2- agonists + inhaled anticholinergics, LABAs, theophylline, other xanthines, parenteral or oral corticosteroids
B2346, 2008/ FELDMAN, 2010	12 week RCT, PC, DB, MC, DD	Indacaterol; 150 μg; OD (n = 211) vs. Placebo (n = 205)	# centres NR/ USA, Australia/ New Zealand, Belgium	FEV1 ≥ 30 % and <80 %; FEV1/FVC < 70 %; reversible and non-reversible patients included; excluded if hospitalisation 6 wks prior	ICS monotherapy	Tiotropium, short acting anti-cholinergics, fixed combinations of β2-agonists and ICS or β 2-agonists and inhaled anticholinergics, LABAs, and other SABAs, theophylline, other xanthines, parenteral or oral corticosteroids
B2354, 2010	12 week RCT, PC, DB, MC	Indacaterol; 75 μg; OD (n = 163) vs. Placebo (n = 160)	# centres NR/ USA	FEV1 ≥ 30 % and <80 %; FEV1/FVC < 70 %; Excluded if exacerbation in 6 wks prior	Antibiotics or oral corticosteroids for exacerbation; ICS monotherapy	LABAs; anticholinergic
B2355, 2010	12 week RCT, PC, DB, MC	Indacaterol; 75 μg; OD (n = 159) vs. Placebo (n = 159)	# centres NR/ USA	FEV1 ≥ 30 % and <80 %; FEV1/FVC < 70 %; Excluded if exacerbation in 6 wks prior	Antibiotics or oral corticosteroids for exacerbation; ICS monotherapy	LABAs; anticholinergic

^1^RCT = randomized clinical trial; PC = placebo-controlled; DB = double-blind; MC = multi-centre; DD = Double dummy; NR = not reported; ^2^UK = United Kingdom; USA = United Sates of America; S. America = South America; ^3^FEV1 = forced expiratory volume in 1 second; FVC = Forced vital capacity; wks = weeks; ^4^ICS = inhaled corticosteroid; LABA = long-acting beta-agonist; h = hour.

**Table 2:** Key patient characteristics at baseline for all studies

**Author**	**Treatment**	**% Male**^**1**^	**Age (sd or range)**^**2**^	**% Current smoker**^**3**^	**% Severe**^**4**^	**% on ICS**^**5**^	**FEV**_**1**_**mean L (sd)**^**6**^	**Reversibility post-SABA (sd)**^**7**^
MOITA, 2008	Tiotropium; 18 μg; OD	NR	NR	28 %	NR	NR	NR	NR
	Placebo	NR	NR	25 %	NR	NR	NR	NR
VERKINDRE, 2006	Tiotropium; 18 μg; OD	94 %	61 (9.5)	24 %	95 %	NR	1.05 (0.4)	NR
	Placebo	94 %	60 (10.2)	33 %	94 %	NR	1.08 (0.3)	NR
COVELLI, 2005	Tiotropium; 18 μg; OD	66 %	66 (8.9)	40 %	77 %	54 %	1.06 (0.4)	NR
	Placebo	49 %	63 (9.2)	37 %	80 %	58 %	0.99 (0.4)	NR
CASABURI, 2000	Tiotropium; 18 μg; OD	67 %	65 (8.6)	NR	79 %	NR	1.04 (0.4)	NR
	Placebo	63 %	66 (9.0)	NR	80 %	NR	1.00 (0.4)	NR
CASABURI, 2002	Tiotropium; 18 μg; OD	67 %	65 (9.0)	NR	79 %	44 %	1.04 (0.4)	NR
	Placebo	63 %	65 (9.0)	NR	80 %	40 %	1.00 (0.4)	NR
NIEWOEHNER, 2005	Tiotropium; 18 μg; OD	98 %	68 (8.7)	29 %	87 %	61 %	1.04 (0.4)	NR
	Placebo	99 %	68 (8.5)	30 %	87 %	58 %	1.04 (0.4)	NR
CHAN, 2007	Tiotropium; 18 μg, OD	59 %	68 (8.7)	32 %	79 %	66 %	0.97 (0.4)	NR
	Placebo	61 %	67 (9.1)	30 %	78 %	71 %	0.96 (0.4)	NR
TONNEL, 2008	Tiotropium; 18 μg: OD	87 %	65 (9.7)	24 %	58 %	38 %	1.38 (0.4)	NR
	Placebo	85 %	64 (10.1)	30 %	62 %	36 %	1.35 (0.5)	NR
HANANIA, 2003	Salmeterol; 50 μg; BID	58 %	64 (42–87)	51 %	75 %	0 %	1.25 (0.4)	NR
	Placebo	68 %	65 (40–81)	47 %	75 %	0 %	1.29 (0.4)	NR
MAHLER, 2002	Salmeterol; 50 μg; BID	64 %	64 (40–84)	46 %	NR	31 %	1.23 (NR)	NR
	Placebo	75 %	64 (44–90)	54 %	NR	18 %	1.31 (NR)	NR
VAN RUTTEN, 1999	Salmeterol; 50 μg; BID	89 %	65 (5.8)	NR	73 %	81 %	1.30 (0.4)	NR
	Placebo	86 %	63 (7.4)	NR	77 %	76 %	1.30 (0.4)	NR
CELLI, 2003	Salmeterol; 50 μg; BID	80 %	64 (8.7)	NR	73 %	NR	1.30 (0.5)	NR
	Placebo	71 %	65 (8.7)	NR	69 %	NR	1.35 (0.5)	NR
GROSS, 2008	Formoterol; 12 μg; BID	54 %	63 (9.4)	54 %	70 %	23 %	1.30 (0.4)	15 % (NR)
	Placebo	57 %	64 (9.2)	54 %	64 %	19 %	1.36 (0.5)	11 % (NR)
ROSSI, 2002	Formoterol; 12 μg; BID	87 %	63 (NR)	NR	NR	NR	1.36 (NR)	NR
	Placebo	80 %	63 (NR)	NR	NR	NR	1.40 (NR)	NR
DAHL, 2001	Formoterol; 12 μg; BID	74 %	64 (8.8)	46 %	63 %	47 %	1.33 (0.5)	NR
	Placebo	79 %	63 (9.0)	49 %	68 %	54 %	1.29 (0.4)	NR
BRIGGS, 2005	Tiotropium; 18 μg; OD	65 %	64 (8.6)	35 %	86 %	54 %	1.05 (0.4)	14 % (NR)
	Salmeterol; 50 μg; BID	68 %	65 (7.8)	37 %	84 %	47 %	1.04 (0.4)	18 % (NR)
B2334, 2008 DAHL, 2010	Indacaterol; 300 μg; OD	80 %	64 (8.6)	42 %	46 %	56 %	1.48 (0.5)	12 % (13 %)
	Formoterol; 12 μg; BID	80 %	64 (8.5)	41 %	44 %	51 %	1.50 (0.5)	12 % (13 %)
	Placebo	82 %	63 (8.3)	40 %	45 %	52 %	1.52 (0.5)	13 % (13 %)
B2335S, 2008 DONOHUE, 2010	Indacaterol; 150 μg; OD	62 %	63 (9.4)	45 %	38 %	38 %	1.52 (0.5)	16 % (15 %)
	Indacaterol; 300 μg; OD	63 %	63 (9.3)	45 %	38 %	37 %	1.53 (0.5)	15 % (15 %)
	Tiotropium; 18 μg; OD	65 %	64 (8.8)	45 %	43 %	35 %	1.45 (0.5)	16 % (18 %)
	Placebo	61 %	64 (8.9)	46 %	40 %	40 %	1.51 (0.5)	16 % (18 %)
B2336, 2009 KORNMANN, 2010	Indacaterol; 150 μg; OD	72 %	63 (8.7)	46 %	42 %	45 %	1.48 (0.5)	12 % (15 %)
	Salmeterol; 50 μg; BID	75 %	63 (9.2)	46 %	43 %	46 %	1.48 (0.5)	11 % (14 %)
	Placebo	77 %	64 (8.6)	45 %	44 %	40 %	1.46 (0.5)	13 % (16 %)
B2346, 2008 FELDMAN, 2010	Indacaterol; 150 μg; OD	51 %	63 (9.9)	51 %	40 %	29 %	1.50 (0.5)	16 % (17 %)
	Placebo	54 %	63 (9.6)	53 %	38 %	34 %	1.50 (0.5)	17 % (19 %)
B2354, 2010	Indacaterol; 75 μg; OD	55 %	64 (8.3)	44 %	41 %	43 %	NR	15 % (13 %)
	Placebo	54 %	64 (9.4)	44 %	44 %	48 %	NR	17 % (14 %)
B2355, 2010	Indacaterol; 75 μg; OD	52 %	61 (9.8)	58 %	30 %	40 %	NR	18 % (17 %)
	Placebo	56 %	62 (9.9)	60 %	45 %	35 %	NR	16 % (14 %)

^1^ % male = proportion of patients who were male; NR = not reported; ^2^Age (sd or range) = average and standard deviation or age range where reported; SE = standard error; ^3^ % Current smokers = proportion of patients who were current smokers as opposed to ex-smokers; ^4^ % severe (very severe) = proportion of patients who had severe or very severe COPD as classified by GOLD guidelines (as opposed to mild or moderate COPD); ^5^ % on ICS = proportion of patients taking concomitant inhaled corticosteroids during trial period; ^6^FEV_1_ mean L = average forced expiratory volume in 1 second. Postbronchodilator FEV_1_ measured 30 min after salbutamol 400 mg inhalation (INVOLVE, INLIGHT-1/2) or albuterol 360 mg inhalation (INHANCE); ^7^SABA = short-acting β2-agonis. Reversibility was calculated as the difference between the prebronchodilator and postbronchodilator values of FEV_1_ (in litres) as a percentage of the prebronchodilator value.

The studies included a predominantly male population, ranging from 49 % to 99 % (51-82 % in indacaterol trials). The average age varied from 60 years to 68 years (61–64 years in indacaterol trials). Across the treatment arms 24 % to 60 % of patients were current smokers (40 %-60 % in indacaterol trials). Patients with severe or very severe COPD per treatment arm ranged from 30 % to 95 % across the treatment arms (30 % to 46 % in indacaterol trials) and the average FEV_1_ at baseline ranged from 0.96 to 1.53 L (1.45 to 1.53 L in indacaterol trials) with reversibility varying from 11 to 18 % across the treatments where reported (11-18 % range in indacaterol trials). The ICS use depended on whether ICS was included as a treatment arm in the study in some cases. Therefore, the proportion of patients using concomitant ICS varied across the studies from 0 % to 81 % (29 % to 56 % in indacaterol trials).

### Network meta-analysis

Table 3 presents the individual study summary statistics based on the IPD for the six indacaterol studies and Table 4 presents the study-level AD extracted for FEV_1_ and SGRQ. Table 5 presents the network meta-analysis results for all of the treatments compared to placebo with adjustment for covariates based on the IPD and AD analyses. Table 6 presents the results for indacaterol 75 μg versus the alternative treatments using both IPD and AD with adjustment for covariates. Results for the analyses without covariates are presented in Tables S1 and S2, as an Additional file 1.

**Table 3:** Individual patient data results for each study and treatment at baseline and 12 weeks

		**PLBO**		**TIO 18**		**SAL 50**		**FOR 12**		**IND 300**		**IND 150**		**IND 75**	
		**FEV**_**1**_	**SGRQ**	**FEV**_**1**_	**SGRQ**	**FEV**_**1**_	**SGRQ**	**FEV**_**1**_	**SGRQ**	**FEV**_**1**_	**SGRQ**	**FEV**_**1**_	**SGRQ**	**FEV**_**1**_	**SGRQ**
B2334, 2008;	N	371	347					379	359	389	372				
	B	1.3	43.6					1.3	44.3	1.3	44.5				
	sd	0.5	17.8					0.4	17.3	0.4	17.1				
	12 wks	1.4	41.6					1.4	39.1	1.4	38.5				
	sd	0.5	18.5					0.5	18.4	0.5	17.9				
B2335S, 2008;	N	376	347	393	374					389	375	389	368		
	B	1.3	45.7	1.2	44.6					1.2	44.6	1.3	45.4		
	sd	0.5	17.3	0.5	18.1					0.5	18.7	0.5	19.1		
	12 wks	1.3	42.7	1.4	41.0					1.4	39.5	1.5	39.9		
	sd	0.5	18.3	0.5	18.4					0.5	18.9	0.5	19.6		
B2336, 2009	N	316	294			316	300					320	309		
	B	1.3	43.6			1.3	43.2					1.3	43.6		
	sd	0.5	17.8			0.5	18.5					0.5	18.7		
	12 wks	1.3	42.4			1.4	37.7					1.5	35.9		
	sd	0.5	19.6			0.5	18.5					0.6	19.4		
B2346, 2008	N	189	187									201	199		
	B	1.3	48.7									1.3	50.1		
	sd	0.6	18.9									0.6	18.9		
	12 wks	1.4	47.6									1.5	43.9		
	sd	0.6	19.2									0.6	19.7		
B23354, 2010	N	148	142											149	147
	B	1.3	49.5											1.3	48.6
	sd	0.5	17.3											0.5	18.7
	12 wks	1.3	47.6											1.4	42.8
	sd	0.5	17.3											0.5	18.2
B23355, 2010	N	150	145											145	148
	B	1.3	50.1											1.4	51.2
	sd	0.5	18.1											0.5	18.1
	12 wks	1.3	49.2											1.5	46.2
	sd	0.5	20.1											0.6	20.0

Mean and standard deviations (sd) presented; IND 75 OD = Indacaterol 75 μg once daily; IND 150 OD = Indacaterol 150 μg once daily; Formoterol 12 μg twice daily; TIO 19 OD = Tiotropium 18 μg once daily; SAL 50 BID = Salmeterol 50 μg twice daily; PLB = Placebo; FEV_1_ = Forced expiratory volume in 1 second; SGRQ = St. George’s Respiratory Questionnaire; TDI = Transition Dyspnea Index; L = Litres; B = Baseline; 12 wks = 12 weeks; N = sample size of data included at 12 weeks. Note: Minor differences in the outcomes compared to the study publications are present due to missing data in the covariate values for this analysis.

**Table 4:** Aggregate data individual study results for FEV_1_ at 12 weeks: difference in change from baseline

		**PLBO**		**TIO 18**		**SAL 50**		**FOR 12**		**IND 300**		**IND 150**		**IND 75**	
		**FEV**_**1**_	**SGRQ**	**FEV**_**1**_	**SGRQ**	**FEV**_**1**_	**SGRQ**	**FEV**_**1**_	**SGRQ**	**FEV**_**1**_	**SGRQ**	**FEV**_**1**_	**SGRQ**	**FEV**_**1**_	**SGRQ**
MOITA, 2008	diff	0	0	0.10											
	se			0.03											
VERKINDRE, 2006	diff	0	0	0.11	−6.50										
	se			0.04	2.90										
COVELLI, 2005	diff	0	0	0.18											
	se			0.04											
CASABURI, 2000	diff	0	0	0.15											
	se			0.01											
CASABURI, 2002	diff	0	0	0.13											
	se			0.02											
NIEWOEHNER, 2005	diff	0	0	0.10											
	se			0.01											
CHAN, 2007	diff	0	0	0.10											
	se			0.02											
TONNEL, 2008	diff	0	0		−3.47										
	se				1.10										
HANANIA, 2003	diff	0	0			0.10									
	se					0.03									
MAHLER, 2002	diff	0	0			0.13									
	se					0.02									
VAN RUTTEN, 1999	diff	0	0				−0.51								
	se						1.66								
CELLI, 2003	diff	0	0				−2.10								
	se						1.28								
GROSS, 2008	diff	0	0					0.08	−3.51						
	se							0.03	1.73						
ROSSI, 2002	diff	0	0					0.04							
	se							0.02							
DAHL, 2001	diff	0	0						−5.10						
	se								1.73						
B2334, 2008;	diff	0	0					0.07	−3.20	0.17	−3.80				
	se							0.02	0.90	0.02	0.90				
B2335S, 2008;	diff	0	0	0.14	−1.10					0.18	−2.50	0.18	−2.80		
	se			0.02	0.86					0.02	0.86	0.02	0.87		
B2336, 2009	diff	0	0			0.11	−4.20					0.17	−6.30		
	se					0.02	1.01					0.02	0.99		
B2346, 2008	diff	0	0									0.13	−4.75		
	se											0.02	1.22		
B23354, 2010	diff	0	0											0.12	−3.80
	se													0.02	1.21
B23355, 2010	diff	0	0											0.14	−3.60
	se													0.02	1.40
BRIGGS, 2005	diff			0.02		0									
	se			0.02											

Diff = Difference versus comparator; NR = Not reported; se = standard error.

**Table 5:** Results of network meta-analysis with adjustment for covariates; treatment effects versus placebo at 12 weeks

	**Trough FEV**_**1**_**L Difference (95%CrI)**		**SGRQ total score Difference**^**1**^**(95%CrI)**	
	**IPD**	**AD**	**IPD**	**AD**
Tiotropium 18	0.13 (0.10; 0.17)	0.13 (0.12; 0.15)	−1.60 (−3.18;-0.05)	−2.99 (−6.48; 0.43)
Salmeterol 50	0.11 (0.07; 0.15)	0.11 (0.09; 0.13)	−3.32 (−5.27;-1.37)	−2.52 (−5.34; 0.44)
Formoterol 12	0.06 (0.03; 0.10)	0.06 (0.04; 0.09)	−2.63 (−4.25;-0.94)	−3.87 (−6.95; -1.16)
Indacaterol 75	0.13 (0.10; 0.16)	0.11 (0.08; 0.14)	−3.02 (−4.87;-1.22)	−4.26 (−7.83; -0.41)

1 = A decrease in SGRQ total score indicates an improvement in health-related quality of life; AD = aggregate data; CrI = 95 % Credibility Interval; FEV_1_ = Forced expiratory volume in 1 second; IPD = Individual patient data; SGRQ = St. George’s Respiratory Questionnaire; IPD covariates: baseline value of outcome; % current smokers; reversibility to short-acting β_2_-agonists (SABA); reversibility to short-acting anticholinergic + covariate interactions with treatments. AD covariates: % current smokers; % severe or very severe COPD; % males; age.

**Table 6:** Results of network meta-analysis with adjustment for covariates; indacaterol 75μg versus alternative treatments at 12 weeks

	**Trough FEV**_**1**_**L**				**SGRQ total score**			
	**IPD**		**AD**		**IPD**		**AD**	
	**Difference (95%CrI)**	**Prob. better**	**Difference (95%CrI)**	**Prob. better**	**Difference (95%CrI)**	**Prob. better**	**Difference (95%CrI)**	**Prob. better**
Tiotropium 18	0.00 (−0.05; 0.04)	44 %	−0.02 (−0.06; 0.01)	12 %	−1.42 (−3.84; 0.97)	88 %	−1.27 (−5.95; 3.74)	72 %
Salmeterol 50	0.02 (−0.03; 0.07)	79 %	0.00 (−0.04; 0.04)	53 %	0.28 (−2.35; 2.97)	42 %	−1.74 (−6.89; 3.54)	77 %
Formoterol 12	0.07 (0.02; 0.11)	>99 %	0.05 (0.01; 0.09)	99 %	−0.40 (−2.90; 2.07)	62 %	−0.38 (−4.99; 4.87)	57 %

AD = aggregate data; CrI = 95% Credibility Interval; FEV_1_ = Forced expiratory volume in 1 second; IPD = Individual patient data; SGRQ = St. George’s Respiratory Questionnaire; IPD covariates: baseline value of outcome; % current smokers; reversibility to short-acting β2-agonists (SABA); reversibility to short-acting anticholinergic + covariate interactions with treatments.AD covariates: % current smokers; % severe or very severe COPD; % males; age.

#### Forced expiratory volume in 1 second (FEV_1_)

**Figure 3:**
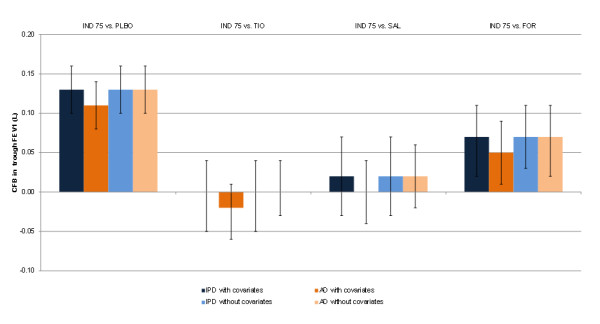
** FEV**_**1**_**results at 12 weeks for the individual patient and aggregate meta-analyses with and without covariates for indacaterol 75 μg versus alternative treatments.** AD = Aggregate data; FOR = Formoterol 12 μg; FEV_1_ = Forced expiratory volume in 1 second; IND 75 = Indacaterol 75 μg; IPD = Individual patient data; SAL = Salmeterol 50 μg; TIO = Tiotropium 18 μg.

All interventions compared were more efficacious than placebo regarding FEV_1_ at 12 weeks in both the IPD and AD analyses (Table 5). Indacaterol 75 μg is expected to result in a comparable FEV_1_ at 12 weeks to tiotropium and salmeterol, and higher FEV_1_ at 12 weeks versus formoterol (Table 6). The results for both IPD and AD are similar and lead to consistent conclusions, although the point estimate for indacaterol 75 μg versus placebo is lower in the AD analysis by a difference of 0.02 L as compared to the IPD analysis (Figure 3).

#### St. George’s respiratory questionnaire (SGRQ)

**Figure 4:**
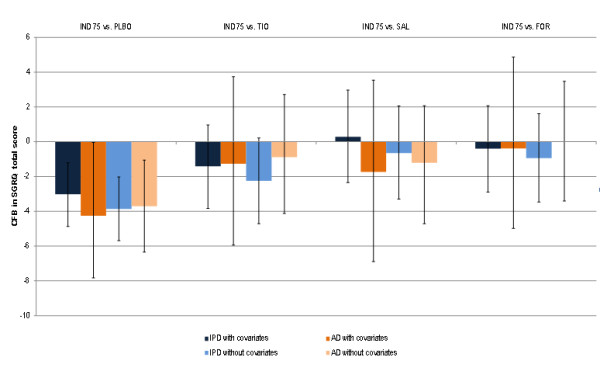
** SGRQ total score results at 12 weeks for the individual patient and aggregate met-analyses with and without covariates for indacaterol 75 μg versus alternative treatments.** AD = Aggregate data; FOR = Formoterol 12 μg; IND 75 = Indacaterol 75 μg; IPD = Individual patient data; SAL = Salmeterol 50 μg; SGRQ = St. George Respiratory Questionnaire; TIO = Tiotropium 18 μg.

For SGRQ an improvement in health-related quality of life is indicated by a decrease in the total score, where a clinically relevant improvement involves a 4 point decrease from baseline [37]. All of the treatments were more efficacious than placebo in terms of SGRQ at 12 weeks, except for tiotropium and salmeterol, where the credible intervals in the AD analysis included zero (tiotropium: -2.99 points improvement versus placebo; 95%CrI −6.48 to 0.43; salmeterol: -2.52 points improvement versus placebo; 95%CrI −5.34 to 0.44) (Table 5). Both IPD and AD results suggest that indacaterol 75 μg is expected to be comparable to all active treatments (Table 6). However, in comparison to salmeterol, the AD results for indacaterol 75 μg were more favourable (−1.74 points; 95%CrI −6.89 to 3.54) than the IPD results (0.28 L points; 95%CrI −2.35 to 2.97), although there was more uncertainty associated with the AD analyses and the point estimates were within the credible intervals of the results for alternative analysis (Figure 4). Results for indacaterol 75 μg in comparison to tiotropium and formoterol were comparable based on IPD and AD analyses, although AD results were slightly less favourable than the IPD results.

## Discussion

The aim of this study was to assess the relative effectiveness of indacaterol 75 μg compared to tiotropium, salmeterol, and formoterol in patients with moderate to severe COPD. Based on the individual patient data and aggregate data analyses, indacaterol 75 μg is expected to be at least as efficacious as formoterol regarding FEV_1_ and comparable to tiotropium and salmeterol. Patients receiving indacaterol 75 μg also experienced a comparable improvement in mean SGRQ total score to those receiving other active treatments.

Although each of the included RCTs provides evidence for the relative efficacy of indacaterol versus an active comparator, none of the studies evaluating indacaterol 75 μg included active comparators. Given that all studies were connected in the network predominantly through placebo, it was possible to indirectly compare the treatments of interest in the network of evidence by synthesizing the results of the RCTs by means of a Bayesian network meta-analysis [15,18]. This framework provides the relative effectiveness for the competing interventions as well as the probability of being the better treatment, which naturally supports decision-making and is intuitive for decision-makers [13,14]. The internal validity of a network meta-analysis is contingent upon the extent of confounding bias due to similarity and consistency violations [16].

Overall, the RCTs were of high quality. A potential limitation of the evidence base is the open-label evaluation of tiotropium in the INHANCE study. Despite this limitation, Donohue et al. [9] reported that the treatment effect of tiotropium compared to placebo was similar to previous results where tiotropium was blinded for trough FEV_1_[35,36,38]. Furthermore, results from a blinded RCT by Buhl et al. 2011 [39] are also comparable to earlier unblinded results from Donohue et al. for the comparison of indacaterol 150ug versus tiotropium. This RCT was not included in the current study as it was published after the search was performed and no individual patient data were available for this study at the time of the analysis.

With a network meta-analysis, randomization only holds within a trial and not across trials. As a result, there is the risk that patients who were assigned the different trials are not comparable. If the distribution of relative treatment-effect modifiers is not similar across trials comparing different interventions in the network of studies, the similarity assumption and consistency assumption in a network meta-analysis is violated and results will be biased [17]. This bias can be limited by adjusting for these differences by incorporating treatment by covariate interactions in the statistical models used.

In the current analysis the degree of heterogeneity between studies included in the AD network meta-analysis was evaluated prior to undertaking the analysis. Differences were identified in terms of the proportion of males, the average age, the proportion of current smokers and the proportion of patients with severe or very severe COPD. As a result the analyses were adjusted for these differences using a constant treatment-by-covariate interaction with reported average values for the patient covariates. Although bias might be reduced, there is always the risk of residual bias with aggregate level data, and the results might still be confounded. In this case, since the IPD and AD results were consistent and the IPD results with and without covariates were comparable; we do not expect that that the network meta-analysis is severely biased due to violations of the similarity or consistency assumptions. Nonetheless, while IPD offers improvement over AD network meta-analysis and might be considered the gold standard to remove bias due to similarity and consistency violations, IPD was only available for a subset of the trials and there is always the risk of residual confounding due covariates not measured in the RCTs. Also, in the current analysis the IPD and AD analyses were evaluated separately. However, it is recommended to perform a network meta-analysis combining the results of these six IPD studies with other AD evidence. Recent simulations have shown that adding IPD to AD studies in a network meta-analysis can dramatically improve precision of the effect estimates [5].

For the AD analyses, random effect models were consistently presented to account for the between study heterogeneity. However, in the case of the IPD analyses, fixed effect models were used given the limited number of studies to estimate the between study heterogeneity. The fixed and random effects models had similar results as indicated by the DIC. The DIC for the random effects model using an uninformed prior has a slightly higher DIC than the fixed effect model. Based on this it can be argued that the fixed effect models are appropriate, especially given that model diagnostics suggest the random effects model using an uninformative prior did not converge.

The outcomes in this study are considered relevant to treatments for COPD. FEV_1_ was the primary endpoint in all of the studies and is also required from a regulatory perspective. Spirometry reflects an important prognostic factor that is used to define severity for COPD, which is considered the most reproducible and objective measurement of airflow limitation available [1]. Lung function and symptoms are the worst in the early morning and therefore affect patient functionality and daily activities [40], while SGRQ represents a key patient reported outcome that provides direct insight into the overall health status of patients. Moreover, the improvements in trough FEV_1_ associated with indacaterol 75 μg (0.13 L) relative to placebo based on the IPD analysis can be considered clinically relevant according to the threshold of 0.12 L pre-specified in the RCTs [41].

Although the current network meta-analysis focused on lung function and overall health status, identification of the ‘best’ or most appropriate treatment cannot be made on the basis of efficacy endpoints alone. To inform health care decision making for clinical treatment guidelines and reimbursement policies, the efficacy findings must be interpreted in light of safety profile of the compared interventions and convenience. Compared to the twice daily dosing required for salmeterol and formoterol, the once daily regimen for indacaterol may improve adherence in clinical practice [42], which has been reported to range from rates as low as 10 % to 40 % for COPD medication [42-45].

In conclusion, based on a synthesis of currently available RCT evidence as well as an individual patient data network meta-analysis of six RCTs, indacaterol 75 μg is expected to be at least as efficacious as formoterol and comparable to tiotropium and salmeterol regarding FEV_1_. Furthermore, indacaterol 75 μg shows a comparable level of improvement in health-related quality of life to tiotropium, salmeterol, and formoterol, as measured by the SGRQ.

## Competing interests

This study was funded by Novartis.

## Author’s contributions

SC and JJ contributed to the analysis and all authors participated in the systematic review and writing. All authors read and approved the final manuscript.

## Pre-publication history

The pre-publication history for this paper can be accessed here:

http://www.biomedcentral.com/1471-2466/12/29/prepub

## Supplementary Material

Additional file 1 **Table S1.** Results of network meta-analysis without adjustment for covariates; treatment effects versus placebo at 12 weeks. **Table S2.** Results of network meta-analysis without adjustment for covariates; indacaterol 75μg versus alternative treatments. Click here for file
