# Supplementary material for: Efficacy of once-daily indacaterol 75 μg relative to alternative bronchodilators in COPD: A study level and a patient level network meta-analysis
Source: BMC Pulm Med. 2012 Jun 25;12:29. doi: 10.1186/1471-2466-12-29 (PMC3512498; doi:10.1186/1471-2466-12-29)
Supplement: Additional file 1 — Table S1. Results of network meta-analysis without adjustment for covariates; treatment effects versus placebo at 12 weeks. Table S2. Results of network meta-analysis without adjustment for covariates; indacaterol 75μg versus alternative treatments. [file 1471-2466-12-29-S1.docx]

Table S1. Results of patient level network meta-analysis without adjustment for covariates; treatment effects versus placebo at 12 weeks

|  | **Trough FEV_1_ L Difference (95%CrI)** | | **SGRQ total score Difference (95%CrI)** | |
| --- | --- | --- | --- | --- |
|  | IPD | AD | IPD | AD |
| Tiotropium 18 | 0.13 (0.10; 0.17) | 0.13 (0.11; 0.14) | -1.62 (-3.21; 0.01) | -2.82 (-5.19; -0.82) |
| Salmeterol 50 | 0.11 (0.07; 0.15) | 0.11 (0.09; 0.13) | -3.23 (-5.20; -1.29) | -2.49 (-4.52; -0.25) |
| Formoterol 12 | 0.06 (0.03; 0.09) | 0.06 (0.03; 0.09) | -2.94 (-4.67; -1.21) | -3.68 (-5.93; -1.54) |
| Indacaterol 75 | 0.13 (0.10; 0.16) | 0.13 (0.10; 0.16) | -3.87 (-5.69; -2.03) | -3.71 (-6.35; -1.06) |

AD=aggregate data; CrI=95% Credibility Interval; FEV_1_= Forced expiratory volume in 1 second; IPD=Individual patient data; SGRQ= St. George’s Respiratory Questionnaire;

Table S2. Results of patient level network meta-analysis without adjustment for covariates; indacaterol 75µg versus alternative treatments

|  | **Trough FEV_1_ L** | | | | **SGRQ total score** | | | |
| --- | --- | --- | --- | --- | --- | --- | --- | --- |
|  | IPD | | AD | | IPD | | AD | |
|  | Difference (95%CrI) | Prob. better | Difference (95%CrI) | Prob. better | Difference (95%CrI) | Prob. better | Difference (95%CrI) | Prob. better |
| Tiotropium 18 | 0.00  (-0.05; 0.04) | 43% | 0.00  (-0.03; 0.04) | 60% | -2.25  (-4.71; 0.22) | 96% | -0.89  (-4.13; 2.72) | 71% |
| Salmeterol 50 | 0.02  (-0.03; 0.07) | 81% | 0.02  (-0.02; 0.06) | 83% | -0.65  (-3.30; 2.06) | 68% | -1.22  (-4.72; 2.07) | 78% |
| Formoterol 12 | 0.07  (0.03; 0.11) | >99% | 0.07  (0.02; 0.11) | >99% | -0.94  (-3.47; 1.61) | 76% | -0.02  (-3.41; 3.47) | 51% |

AD=aggregate data; CrI=95% Credibility Interval; FEV_1_= Forced expiratory volume in 1 second; IPD=Individual patient data; SGRQ= St. George’s Respiratory Questionnaire;
